# Haematological, Biochemical and Antioxidant Changes in Wistar Rats Exposed to Dichlorvos Based Insecticide Formulation Used in Southeast Nigeria

**DOI:** 10.3390/toxics4040028

**Published:** 2016-11-29

**Authors:** Kingsley C. Kanu, Solomon N. Ijioma, Odudu Atiata

**Affiliations:** 1 Department of Environmental Management and Toxicology, Michael Okpara University of Agriculture, PMB 7267 Umudike, Nigeria; oduduatiata@gmail.com; 2 Department of Physiology and Pharmacology, College of Veterinary Medicine Michael Okpara University of Agriculture, PMB 7267 Umudike, Nigeria; ijiomasolo@yahoo.co.uk

**Keywords:** dichlorvos toxicity, hepatotoxic, haematological alteration, antioxidant defense, renal toxicity

## Abstract

The indiscriminate use of pesticide is a treat to non-target organisms. This study evaluates the haematological and biochemical changes induced by inhalation of local Nigerian dichlorvos insecticide on rats. The rats were randomly assigned to a control group which received only food and water and a test group which, in addition to food and water, was exposed to the pesticide for a period of 4 h daily for 28 days, after which exposure was discontinued for seven days. Five animals were sacrificed from each group on days 1, 7, 14, 21, 28 and 35, and blood was collected by cardiac puncture for haematological, biochemical and antioxidant analysis. Results obtained showed lowered values of red blood cell count (RBC), packed cell volume (PCV), haemoglobin, mean cell haemoglobin (MCH) and mean cell haemoglobin concentration (MCHC) (*p* < 0.05) with increased white blood cell count (WBC) and platelet counts after day 14 when compared to the control group. Liver enzymes aspartate amino transaminase (AST) and alanine amino transaminase (ALT) were higher in the exposed rats compared to the control group (*p* < 0.05). Urea and creatinine concentrations increased significantly after day 1 and at day 28, while superoxide dismutase (SOD), gluthathione (GSH) and catalase (CAT) activity increased significantly compared to the control after day 1, day 14 and day 21, respectively. The RBC, PCV and haemoglobin values of all exposed rats were restored to normal following withdrawal of the pesticide, though AST, ALT, urea, creatinine and, glutathione values remained significantly high compared to the control. Inhalation of the local insecticide is toxic to the blood, liver and kidney of laboratory rats and may be deleterious to human health following long-term exposure.

## 1. Introduction

Pesticides are ubiquitous and global statistics have revealed increasing use of these chemicals for the control of pests [1]. Pesticides are usually applied directly to control pests present in both the indoor and outdoor environment where humans spend a considerable amount of time. Gale et al. [2] in their study identified more than 400 chemicals including pesticides in the household air of 52 homes on the border between Arizona and Mexico. Common experience shows that humans spend a greater percentage of their time indoors (60%–90%) than outdoors [3] in [4], hence significant exposure to pesticides can occur in the home [5]. Indoor air pollution poses high risks to humans, especially susceptible groups such as infants, the elderly and pregnant women [6].

The World Health Organization (WHO) recommends indoor residual spraying of insecticide to control the malaria vector [7]. In Nigeria, locally formulated insecticides containing dichlorvos as the active ingredient are widely employed in the control of mosquitoes, cockroaches and termites. Otapiapia is the local parlance used to describe such formulations. The concentration of dichlorvos in otapiapia varies from 1% to 10% [8,9]. The widespread use of otapiapia in homes gained acceptance due to its availability, efficacy and affordability [10]. A locally formulated product was reported to contain toluene, cumene, 1,2,3-Trimethyl-benzene, decane, undecane, dodecane, 11, 12-dibromo-tetradecan-1-ol acetate, and pentadecane, with dichlorvos being the major component [11].

Several studies have been conducted to evaluate the effect of dichlorvos insecticide on blood and liver via oral exposure. It is pertinent to assess the effects of inhalation of commonly sold local pesticides and to determine if the effects can be ameliorated by withdrawal. Consequently, the objective of the current study was to observe the effects of inhalation of a locally formulated pesticide containing dichlorvos as the active ingredient on the biochemical, antioxidant and haematological profile of albino rats following 28 days of exposure to the agent and to observe changes in the same parameters after withdrawal of the agent.

## 2. Materials and Methods

### 2.1. Study Design

The trade name of the local insecticide used in this study is “Original Uduotali Super Insecticide” a product of Vatudas factory. The insecticide which contains dichlorvos as active ingredient was purchased from a road side hawker in Umuahia, Abia State, Nigeria. Male Albino rats weighing between 100 and 150 g used for the study were procured from the laboratory animal production unit of the College of Veterinary Medicine, Michael Okpara University of Agriculture, Umudike, Nigeria and acclimatized for two weeks in the laboratory prior to the start of the experiment. Animals were fed with rat chore (vital grower mash) produced by Grand Cereal Limited, Jos, Plateateu State, Nigeria before and during the experiment. The rats were randomly assigned to a control group and test group with each group divided into six sub-groups based on sampling intervals (day 1, 7, 14, 21, 28 and 35). Test group was exposed to pesticide by inhalation while the control group was not. The exposed rats were placed in a poorly ventilated cage and exposed daily to the pesticide by inhalation for 4 h. Then 5 mL of the pesticide was soaked in cotton wool and placed inside the cages using a container that prevents the rats from ingesting them. At the predetermined intervals, samples were randomly collected from both the control and test sub-groups for analysis. Exposure was discontinued after day 28 and seven days later samples were also collected for analysis. The rats were handled in accordance with the international principles on the care and use of experimental animals [12] and guidelines of Department of Physiology, Pharmacology, Biochemistry and Animal Health ethics committee-VPP/EC/2016/008 approved on 3 March 2016.

### 2.2. Sample Collection, Haematological, Biochemical and Antioxidant Assessment

Five rats each were randomly selected from the sub-groups for blood collection by cardiac puncture. The blood samples were collected into K_3_EDTA and plain bottles before analysis. The parameters analyzed included haemoglobin (Hb) concentration, packed cell volume (PCV), red blood cell count (RBC), white blood cell count (WBC), platelet (PLT), Mean cell volume (MCV), mean cell haemoglobin (MCH) and Mean cell haemoglobin concentration (MCHC). These parameters were obtained at once for each blood sample using automated haematology Analyzer ERMA (model pce 210) made in Japan. Biochemical parameters including alanine amino transferase (ALT), aspartate amino transferase (AST), urea, creatinine, were determine using commercial kits and following standard procedures outlined by the producer, Randox Laboratories, UK. Antioxidants assayed include superoxide dismutase (SOD), glutathione (GSH) and Catalase (CAT). SOD activity was determined according to the method described by Fridovich [13]. CAT was assayed spectrophotometrically according to the method of Aebi et al. [14], while reduced glutathione was determined by the method of Jollow et al. [15].

### 2.3. Data Analysis and Statistical Procedures

The mean values of the haematological, biochemical and antioxidant activity of the test group were compared with control groups using *t*-tests while one-way ANOVA was used to compare means across the intervals. Turkeys test post hoc test was used to obtain the specific significant differences among the sampling intervals. Analysis was computed with SPSS for windows, version 16.0. Chicago, SPSS Inc.

## 3. Results

### 3.1. Effects of Exposure to Local Pesticide on Haematological Parameters

The result of the effect of the pesticide formulation on haematological indices is presented in Table 1 and Table 2. RBC, PCV, and haemoglobin were significantly lower (*p* < 0.05), while the WBC count was significantly higher in the exposed rats compared to the controls after day 1, 14, 21 and 28. The platelet count was higher than that of the controls (*p* < 0.05) at day 21 and 28. The trend showed that the RBC, PCV, haemoglobin and WBC of exposed rats increased as the exposure days increased and the significant intervals were day 1 to 7 and day 14 to 21 for RBC; day 1 to 7, day 7 to 14 and day 21 to 28 for haemoglobin; and day 1 to 7 and day 7 to 14 for WBC. Also, the platelet count deceased as the exposure days increased and it was significant between day 1 and 7 (*p* < 0.05). MCH and MCHC were significantly lower than those of the control at day 21 (Table 2). Discontinuation of exposure after day 28 for seven days caused a significant increase in the RBC, PCV and haemoglobin of the exposed rats, and although RBC, PCV, haemoglobin and WBC were restored in the exposed rats, only haemoglobin was significantly higher than that of the control (Table 1).

### 3.2. Effects of Exposure to Local Pesticide on Biochemical Parameters

The effect of the pesticide formulation on biochemical indices is presented in Table 3. Liver enzymes AST and ALT were significantly higher in the exposed rats compared to the control group at day 7, 14, 21, and 28 (*p* < 0.05), while urea and creatinine increased significantly at day 1 and day 28. The trend shows an increase in AST after day 1 up to day 21, which was significantly different between day 1 to 7 and day 14 to 21, while ALT increased significantly from day 1 to 7. Discontinuation of exposure after day 28 did not result in the reversal of the effects of the local pesticides as AST, ALT, urea and creatinine were all significantly higher in the exposed rats compared to the controls (Table 3).

### 3.3. Effects of Exposure to Local Pesticide on Antioxidants

**Figure 1:**
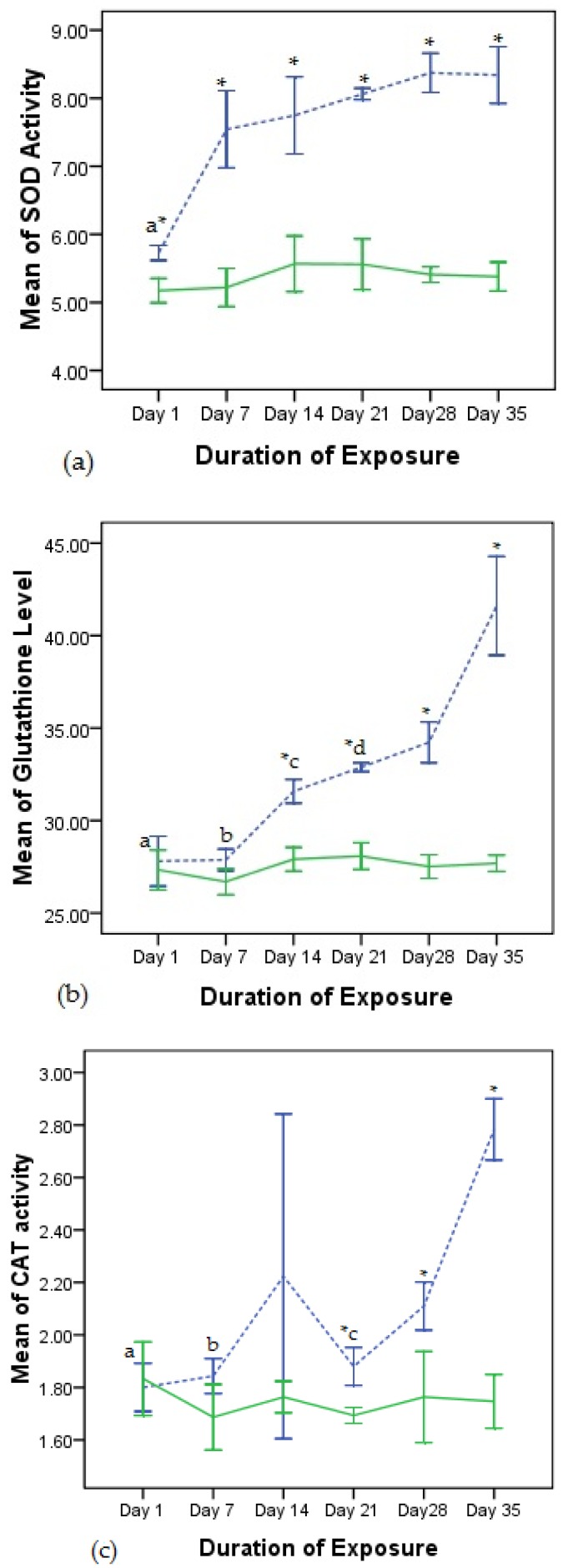
Line graph of antioxidant activity in exposed rats compared to controls, data represent the mean and standard deviation of five rats, broken line indicates exposed rats while the continuous line indicates control; * indicates significant difference between exposed rats and control (*p* < 0.05): (**a**) Superoxide dismutase SOD activity (U/L), ‘a’ indicates significant difference compared to day 7, 14, 21, 28 and 35; (**b**) Glutathione activity (U/L), ‘a’ and ‘b’ indicate significant differences compared to day 14, 21, 28 and 35, ‘c’ and ‘d’ indicate significant differences compared to day 35 ; (**c**) Catalase activity (U/L), ‘a’, b’ and ‘c’ indicate significant differences compared to day 35.

The trend of the activity of superoxide dismutase (SOD), glutathione (GSH) and catalase (CAT) in the exposed rats compared to the controls is presented in Figure 1. SOD was induced (*p* < 0.05) in the exposed rats compared to the controls from day 1 to day 28 and though it increased as the exposure increased, only the increase from day 1 to day 7 was significant (Figure 1a). Glutathione activity was significantly induced at day 14, 21 and 28 in exposed rats compared to the control group, while the increase from day 7 to day 14 in the exposed rats was significant (Figure 1b). CAT was induced (*p* < 0.05) at day 21 and 28 in the exposed group compared to the control (Figure 1c). Glutathione activity increased significantly after the discontinuation of exposure by day 28.

## 4. Discussion

Following inhalation, toxicants are transported by the blood to various organs including the liver and kidney where they may eventually cause harmful effects. Blood can act as a pathological and physiological indicator of animal health [16]. In this study, a significant decrease in the RBC, PCV, and haemoglobin levels at day 1, 14, 21 and 28 is indicative of anaemia. This is in agreement with earlier findings of Holy et al. [17] after intraperitoneal administration of dichlorvos. Celik et al. [18], however, did not obverse any alteration in RBC, PCV and haemoglobin values of rats exposed to 5 and 10 ppm of dichlorvos via ingestion. Red blood cell indices reflect the size (MCV) and level of haemoglobin content (MCH and MCHC) of the red blood cells and aid in the diagnosis of the cause of anaemia. The red blood cells of the exposed rats were normocytic as their MCV values did not differ significantly from the controls, while the cells were hypochromic at day 21 and normochromic at other days as the MCH and MCHC values differed significantly in day 21. The MCV, MCH and MCHC values suggest that normocytic normochromic anaemia may have occurred at day 1, 7, 14 and 28, while normocytic hypochromic anaemia occurred at day 21. This could be attributed to the increased destruction of the red blood cells by the dichlorvos beyond the production capacity of the bone marrow [17] and the fall in the levels of the body iron content. The anaemia caused by dichlorvos was, however, ameliorated after the withdrawal of the pesticide after seven days, resulting in the restoration of RBC, PCV and haemoglobin recorded by this study. The increase in WBC (*p* < 0.05) at day 14, 21 and 28 is indicative of leukocytosis, while the increase in platelet count at day 21 and 28 possibly suggests secondary thrombocytosis. This is in consonance with the earlier observations of Holy et al. [17] and Celik et al. [18]. Leukocytosis and secondary thrombocytosis may be caused by benign conditions such infections, inflammation-tissue necrosis, stress and hemolytic anaemia [19,20].

Akomas et al. [21] reported the development of hypochronic anaemia due to a fall in the iron content of the body resulting from oxidative stress. Exposure to dichlorvos induces oxidative stress by generation of reactive oxygen species (ROS) [22]. Oxidative stress occurs when the production of harmful molecules called free radicals is beyond the protective capability of the antioxidant defenses [23]. Disruption of the activities of antioxidants suggests alteration of the oxidative state of blood cells by the pesticide formulation. The increase in SOD, CAT and GSH levels recorded in this study is consistent with earlier findings [24,25,26]. However, a decrease in these parameters following ingestion and intravenous injection of dichlorvos has also been reported [27,28]. Withdrawal of the pesticide for seven days did not alter the trend of the activities of SOD, CAT and GSH in the exposed rats as they were observed to be significantly higher compared to the controls, although GSH increased significantly compared to day 28.

The liver is the organ most commonly involved in the metabolism of endogenous and foreign compounds. Blood is transported to the liver through the portal vein which carries blood containing digested nutrients from the gastrointestinal tract and the hepatic artery which carries oxygenated blood from the lungs [29]. Liver enzymes AST and ALT are frequently used as biomarkers of liver injury because they are released by hepatocytes into the extracellular space [30]. The significant increase in AST and ALT recorded by this study indicates that the pesticide formulation is hepatoxic. This is agreement with the earlier observations of the effect of dichlorvos by Celik et al. [18] and Garba et al. [9]. Serum creatinine and blood urea have typically been used to diagnose kidney injury [31]. The significant increase in urea and creatinine observed suggests that the pesticide is possibly nephrotoxic. Previous studies have equally observed an increase in creatinine [28,32] and blood urea [28] as the duration of the exposure increased. Again, withdrawal of the pesticide after day 28 did not ameliorate the damage to the hepatocytes and kidney as serum AST, ALT, urea and creatinine were all significantly higher in the exposed rats compared to the controls. 

## 5. Conclusions

The exposure of rats to local dichlorvos-based insecticide lowered haematological values, raised the levels of liver and kidney biomarkers and altered antioxidants in treated rats with recovery in only haematological parameters following withdrawal. With the high probability that the reported effects might be due to organic solvents which have a higher vapor pressure than dichlorvos, there is a need for further research on the safety of local insecticide formulation produced in southeast Nigeria.

## Figures and Tables

**Table 1:** Mean haematological indices as compared to controls.

Duration of Exposure	Sub-Group	RBC (×10^12^/L)	PCV (%)	Hb (g/dL)	WBC (×10^9^/L)	Platelets Count (×10^9^/L)
Day 1	Control A	6.33 ± 0.31	41.13 ± 0.58	12.47 ± 0.58	18.9 ± 1.00	293 ± 1.00
	Test A	5.28 ± 0.14 *^a+^	34.8 ± 1.04 *^a+^	10.27 ± 0.29 *^a+^	20.13 ± 2.89 *^a+^	233.3 ± 4.04 *^a+^
Day 7	Control B	6.6 ± 0.35	37.67 ± 0.58	11.13 ± 0.12	5.4 ± 0.34	360.67 ± 0.58
	Test B	6.01 ± 0.01 ^+^	37.2 ± 0.52 ^+^	11.17 ± 0.23 ^b+^	6.1 ± 2.8 ^b+^	415.67 ± 77.36 ^+^
Day 14	Control C	7.03 ± 0.02	41.47 ± 0.40	12.07 ± 0.06	10.8 ± 0.69	386.67 ± 1.16
	Test C	5.63 ± 0.47 *^b+^	35.9 ± 1.56 *^+^	10.47 ± 0.06 *^c+^	14.9 ± 1.56 *	467.33 ± 113.74 ^+^
Day 21	Control D	7.25 ± 0.05	42.34 ± 0.04	12.46 ± 0.05	12.4 ± 0.35	281.33 ± 1.16
	Test D	6.26 ± 0.10 *	36.85 ± 1.17 *^+^	10.42 ± 0.23 *^d+^	16.85 ± 1.87 *	393.33 ± 25.40 *
Day28	Control E	7.33 ± 0.017	44.03 ± 0.84	13.29 ± 0.10	13.46 ± 0.40	349.67 ± 4.5
	Test E	6.45 ± 0.13 *^+^	38.93 ± 0.81 *^+^	11.23 ± 0.06 *^+^	15.13 ± 0.16 *	391.33 ± 1.16 *
Day 35 (seven days withdrawal)	Control F	7.13 ± 0.02	42.12 ± 0.10	12.23 ± 0.12	18.13 ± 0.12	263.67 ± 1.16
	Test F	7.25 ± 0.10	45.76 ± 1.6	12.84 ± 0.19 *	21.23 ± 4.27	236.67 ± 28.87

The results are mean ± standard deviation (SD) for five rats. * significantly different compared to control (*p* < 0.05); within the test group, + significantly different from day 35; RBC: ‘a’ significantly different compared to day 7, 21 and 28, while ‘b’ significantly different compared to day 21 and 28; PCV: ‘a’ significantly different from day 28; Hb: ‘a’ significantly different compared to day 7 and 28, ‘b’ significant different compared to day 14 and 21, ‘c’ and ‘d’ significant different compared to day 28; WBC: ‘a’ significantly different from day 7, 14, 21 and 28, ‘b’ significant different from day 14, 21 and 28; platelet count; ‘a’ indicates significantly different from day 7, 14, 21 and 28.

**Table 2:** Mean red blood cell indices as compared to controls.

Duration of Exposure	Sub-group	MCV (fL)	MCH (Pg)	MCHC (g/dL)
Day 1	Control A	65.08 ± 4.09	19.72 ± 1.48	30.31 ± 1.26
	Test A	65.87 ± 1.65 ^a^	19.43 ± 0.2 ^a^	29.50 ± 0.05
Day 7	Control B	57.20 ± 3.62	16.90 ± 1.02	29.56 ± 0.15
	Test B	61.86 ± 0.98	18.57 ± 0.35 ^b^	30.03 ± 1.03
Day 14	Control C	59.01 ± 0.38	17.17 ± 0.12	29.10 ± 0.37
	Test C	63.87 ± 2.48 ^b^	18.67 ± 1.59 ^c^	29.20 ± 1.40
Day 21	Control D	58.38 ± 0.33	17.18 ± 0.04	29.43 ± 0.10
	Test D	58.86 ± 0.88	16.65 ± 0.08 *	28.28 ± 0.28 *
Day28	Control E	60.07 ± 1.23	18.13 ± 0.16	30.18 ± 0.35
	Test E	60.33 ± 0.01	17.41 ± 0.45	28.86 ± 0.76
Day 35 (seven days withdrawal)	Control F	59.10 ± 0.05	17.16 ± 0.11	29.04 ± 0.20
	Test F	63.18 ± 3.07	17.72 ± 0.02 *	28.10 ± 1.44

The results are mean ± SD for five rats. * Significantly different compared to control (*p* < 0.05); within the test group, MCV: ‘a’ significantly different compared to day 21 and 28, ‘b’ significantly different compared to day 21; MCH: ‘a’ significantly different compared to day 21 and 28, ‘b’ and ‘c’ significantly different compared to day 21.

**Table 3:** Mean biochemical indices as compared to controls.

Duration of Exposure	Sub-Group	AST (μ/L)	ALT (μ/L)	Urea (mg/dL)	Creatinine (mg/dL)
Day 1	Control A	29 ± 2.65	31.67 ± 4.51	20.28 ± 1.89	1.17 ± 0.09
	Test A	30.33 ± 8.74 ^a+^	37.67 ± 0.58 ^a+^	24.4 ± 0.81 *^a+^	1.51 ± 0.09 *
Day 7	Control B	30.67 ± 7.37	40.33 ± 6.66	23 ± 1.39	1.19 ± 0.17
	Test B	51.67 ± 1.53 *^b+^	72 ± 5.29 *	25.55 ± 2.37	1.37 ± 0.22
Day 14	Control C	32.33 ± 3.21	35 ± 1.73	23.83 ± 2.34	1.23 ± 0.03
	Test C	59.33 ± 4.04 *^c^	63 ± 3.0 *	29.98 ± 5.5	1.25 ± 0.05
Day 21	Control D	36.33 ± 3.51	37.33 ± 1.15	22.27 ± 1.45	1.17 ± 0.07
	Test D	76.33 ± 3.79 *	64.67 ± 4.04 *	24.55 ± 1.45 ^+^	1.46 ± 0.19
Day28	Control E	37 ± 6.25	32 ± 1.0	22.85 ± 1.28	1.15 ± 0.06
	Test E	67.33 ± 3.06 *	65.33 ± 5.03 *	34.96 ± 3.46 *	1.48 ± 0.17 *
Day 35 (seven days withdrawal)	Control F	32.67 ± 4.51	35.67 ± 3.21	22.01 ± 1.39	1.2 ± 0.05
	Test F	66 ± 3.61 *	63 ± 1.0 *	35.66 ± 6.12 *	1.56 ± 0.1 *

* indicates significantly different compared to control (*p* < 0.05); within the test group + significantly different from day 35; AST: ‘a’ significantly different compared with day 7, 14, 21 and 28, ‘b’ significantly different compared to day 21 and 28, ‘c’ significantly different compared with day 21; ALT: ‘a’ significantly different compared to day 7, 14, 21 and 28; Urea: ‘a’ significantly different compared with day 28.
